# Complete Genome Sequence of *Pseudomonas aeruginosa* PA1, Isolated from a Patient with a Respiratory Tract Infection

**DOI:** 10.1128/genomeA.01453-15

**Published:** 2015-12-10

**Authors:** Shuguang Lu, Shuai Le, Gang Li, Mengyu Shen, Yinling Tan, Xia Zhao, Jing Wang, Wei Shen, Keke Guo, Yuhui Yang, Hongbin Zhu, Shu Li, Ming Li, Junmin Zhu, Xiancai Rao, Fuquan Hu

**Affiliations:** Department of Microbiology, College of Basic Medical Science, Third Military Medical University, Chongqing, People’s Republic of China

## Abstract

We report the 6,498,072-bp complete genome sequence of *Pseudomonas aeruginosa* PA1, which was isolated from a patient with a respiratory tract infection in Chongqing, People's Republic of China. Whole-genome sequencing was performed using single-molecule real-time (SMRT) technology, and *de novo* assembly revealed a single contig with 396-fold sequence coverage.

## GENOME ANNOUNCEMENT

*Pseudomonas aeruginosa* is a Gram-negative rod-shaped gammaproteobacterium that grows in a wide range of ecological niches, such as soil, marshes, and coastal marine habitats, as well as on plant and animal tissues (1–3). As an opportunistic pathogen, *P. aeruginosa* causes a wide range of syndromes in humans that can vary from local to systemic, and sometimes its infection is life-threatening (4). *P. aeruginosa* is a significant pathogen associated with infections of burn victims, urinary tract infections in catheterized patients, and respiratory tract infections (2, 5). When infecting immunocompromised or cystic fibrosis (CF) patients, *P. aeruginosa* can lead to deadly pneumonia (6, 7). Notably, intrinsic drug resistance of *P. aeruginosa* makes it difficult to treat *P. aeruginosa* infections with antibiotics (8, 9).

As of 20 October 2015, 27 complete genome sequences of different *P. aeruginosa* stains have been released from the GenBank database (10) (http://www.ncbi.nlm.nih.gov/genome/genomes/187). Different *P. aeruginosa* genomes share a remarkable amount of sequence similarity, despite having been isolated from various niches or different clinical origins (11–13). The *P. aeruginosa* pangenome consists of at least 4,000 core genes, approximately 10,000 accessory genes, and 30,000 or more rare genes that are present in only a few strains or clonal complexes (4). These genome sequences have provided insight into virulence, drug resistance, and biofilm formation that are related to the pathogenicity of *P. aeruginosa* (2, 14, 15). However, hitherto the genomic information of *P. aeruginosa* is still very limited for researchers to analyze, compare, and evaluate the characteristics of the species. Thus, more *P. aeruginosa* genome sequences are required to explore potential ways to control this versatile opportunistic pathogen.

*P. aeruginosa* PA1 was originally isolated from a respiratory tract infection patient in Chongqing, China. It has a lytic bacteriophage that belongs to the PaP1-like phage genus (16). The genomic DNA of *P. aeruginosa* PA1 was extracted from the stationary-phase cultures grown in LB broth and purified using the TIANamp bacteria DNA kit (Tiangen Biotech, Beijing, China). PacBio single-molecule real-time (SMRT) sequencing of the PA1 genome was carried out at the Institute of Medicinal Plant Development (IMPLAD) (Beijing, China) using the PacBio RS II Instrument (Pacific Biosciences, Menlo Park, CA, USA) (17, 18). Libraries of 5-kb were constructed and 4 SMRT cells of the libraries were sequenced with 180-min movies. *De novo* assembly was performed using RS_HGAP_Assembly v. 2.0 (19), revealing a single contig with an average sequence coverage of 396-fold. The length of the PA1 genome is 6,498,072 bp, with an average G+C content of 66.35%. Genome annotation of *P. aeruginosa* PA1 was performed through the NCBI Prokaryotic Genome Annotation Pipeline (20) (released 2013) (http://www.ncbi.nlm.nih.gov/genome/annotation_prok/).

### Nucleotide sequence accession number.

The complete genome sequence of *P. aeruginosa* PA1 has been deposited in GenBank under the accession number CP004054.
